# The Association Between Early Postoperative Temperature Trajectories and Severe Acute Kidney Injury After Valvular Heart Surgery: A Retrospective Cohort Study

**DOI:** 10.3390/jcm15051887

**Published:** 2026-03-01

**Authors:** Jin Sun Cho, Sungmin Suh, Jae-Kwang Shim, Hye Sun Lee, Hee Won Choi, Hyejin Yang, Young-Lan Kwak

**Affiliations:** 1 Department of Anesthesiology and Pain Medicine, Yonsei University College of Medicine, Seoul 03722, Republic of Korea; chjs0214@yuhs.ac (J.S.C.); aneshim@yuhs.ac (J.-K.S.); ysheewon15@yuhs.ac (H.W.C.); 2 Anesthesia and Pain Research Institute, Yonsei University College of Medicine, Seoul 03722, Republic of Korea; 3 Department of Anesthesiology and Pain Medicine, Soonchunhyang University Seoul Hospital, Soonchunhyang University College of Medicine, Seoul 04401, Republic of Korea; suh5701@schmc.ac.kr; 4 Biostatistics Collaboration Unit, Department of Research Affairs, Yonsei University College of Medicine, Seoul 03722, Republic of Korea; hslee1@yuhs.ac (H.S.L.);

**Keywords:** acute kidney injury, temperature trajectory, cardiac surgery, hypothermia, postoperative care

## Abstract

**Background/Objectives**: Postoperative body temperature abnormalities are common yet underrecognized, and their association with acute kidney injury (AKI) remains unclear. In this study, we aimed to identify early postoperative temperature trajectories and evaluate their associations with AKI. **Methods:** This retrospective cohort study included 3274 adults undergoing valvular heart surgery with cardiopulmonary bypass. The patients’ temperatures were continuously measured using a pulmonary artery catheter for 12 postoperative hours, and temperature trajectories were identified using data-driven latrend class modeling. The primary outcome was severe AKI (KDIGO stage ≥ 2), and the secondary outcome was non-recovery AKI (≥72 h). Multivariable logistic regression and E-value sensitivity analysis were performed. **Results:** Four distinct temperature trajectories were identified: Class 1 (32.8%), initial normothermia progressing to mild hyperthermia (37.5–38.0 °C); Class 2 (27.4%), mild hypothermia (36.0–36.5 °C) with rapid normalization; Class 3 (24.4%), stable normothermia; and Class 4 (15.4%), lower-range mild hypothermia (35.5–36.0 °C) with delayed recovery. Severe AKI and non-recovery AKI occurred most frequently in Class 4 patients (15.1% vs. 2.9%, 3.9%, and 4.8% in Classes 1–3, *p* < 0.001; 15.1% vs. 1.7%, 4.0%, and 4.4%, *p* < 0.001, respectively). After adjusting for key clinical variables, Class 4 remained independently associated with severe AKI (OR 2.44, 95% CI: 1.69–3.57; E-value 4.33) and non-recovery AKI (OR 2.78, 95% CI: 1.89–4.00; E-value 4.97). **Conclusions:** Early postoperative temperature trajectories were significantly associated with severe AKI, with the highest risks in patients exhibiting lower-range mild hypothermia with delayed recovery. These findings suggest that early postoperative temperature patterns may be useful for risk stratification for severe AKI after cardiac surgery.

## 1. Introduction

Body temperature management is a key component of perioperative care [1] as it is a relatively modifiable factor linked to postoperative prognosis [2]. Perioperative temperature abnormalities are common in cardiac surgery due to factors such as advanced age, comorbidities, extensive surgical exposure, and the cooling–rewarming process during cardiopulmonary bypass [3,4]. While transient temperature abnormalities are common, persistent or uncontrolled deviations in temperature regulation may have important physiological consequences, potentially affecting organ function and postoperative recovery [5,6]. Despite routine monitoring, postoperative temperature management remains largely empirical, and its impact on major complications such as acute kidney injury (AKI) remains incompletely understood [7,8].

AKI is a common and serious complication after cardiac surgery, affecting up to 40% of patients [9]. Although most cases are mild or transient, severe and persistent AKI substantially worsens prognosis, increasing mortality risk up to eightfold [10,11]. Given the lack of widely accessible early predictors of AKI, identifying physiological indicators from routine perioperative monitoring could improve risk stratification and facilitate early intervention.

Existing research on postoperative temperature has relied on static temperature thresholds [7] or predefined ranges [5,6], failing to capture the complexity of thermoregulation dynamics. In contrast, temperature trajectory analysis, which considers patterns, duration, and rates of change over time, may offer a more comprehensive and clinically relevant approach. Moreover, unlike previous studies that relied on peripheral temperature measurements [6,12] or lacked clarity on measurement site in retrospective analyses [5,13], continuous central temperature monitoring using a pulmonary artery catheter may more accurately reflect temperature changes. Accordingly, this retrospective study aimed to identify early postoperative central body temperature trajectories and examine their association with severe AKI following valvular heart surgery.

## 2. Materials and Methods

### 2.1. Study Design and Participants

This retrospective study used data from the electronic medical records of Severance Hospital. The study protocol was approved by the Institutional Review Board and Hospital Research Ethics Committee of Severance Hospital, Yonsei University College of Medicine (4-2024-0771), and it is registered at ClinicalTrials.gov (NCT06647771). A data analysis and statistical plan was written and filed with the IRB before data were accessed. The requirement for written informed consent was waived due to this study’s retrospective nature.

Patients who underwent valvular heart surgery with cardiopulmonary bypass from January 2016 to December 2023 were enrolled in this study. The inclusion criteria were age ≥ 18 years and the surgery being performed primarily for valvular heart disease, including cases with concomitant coronary artery bypass graft surgery, aortic procedures, and most other cardiac procedures. Patients were excluded if they had an implanted ventricular assist device or if they underwent transcatheter valve replacement or combined congenital heart disease surgery.

### 2.2. Data Collection

The retrieved preoperative data included the patients’ demographics, comorbidities, the European System for Cardiac Operative Risk Evaluation (EuroSCORE) II, and medications. Intraoperative data included the type of surgery, emergency status, cardiopulmonary bypass and aortic cross-clamp times, and transfusion of packed erythrocytes. Postoperative data included transfusion of packed erythrocytes, major morbidity endpoints during hospitalization, intensive care unit (ICU) length of stay, hospital length of stay, in-hospital mortality, and 30-day postoperative mortality. Laboratory data included preoperative hemoglobin, platelet count, estimated glomerular filtration rate (eGFR), and serum creatinine, glucose, albumin, and C-reactive protein levels, which were routinely assessed within one month before surgery. Postoperative serum creatinine levels, assessed daily during hospitalization in accordance with our institutional protocol, were also retrieved.

### 2.3. Temperature Management and Measurement

As part of routine clinical practice, a fluid warmer (Level 1; Smiths Medical, Rockland, MA, USA), heated anesthetic circuit (Ace Medical, Seoul, Republic of Korea), forced-air warming blanket (Bair Hugger; 3M, St.Paul, MN, USA), and automated coil-heated mattress(Blanketrol Mega-Circuit; Gentherm Medical, Cincinnati, OH, USA) were used to maintain the patient’s body temperature at 36–37 °C, except during cardiopulmonary bypass. Patients were cooled to 33–34 °C during cardiopulmonary bypass and subsequently rewarmed to a bladder temperature of >36.5 °C before weaning from bypass. Intraoperatively, nasopharyngeal, bladder, and blood temperatures (measured using a pulmonary artery catheter (Swan-Ganz; Edwards Lifesciences, Irvine, CA, USA)) were continuously monitored. A pulmonary artery catheter was routinely inserted for hemodynamic monitoring in all patients undergoing valve surgery, except those undergoing tricuspid valve replacement, and was maintained for 12 h postoperatively. Temperatures were recorded every 15 min intraoperatively and at least once per hour postoperatively. Postoperatively, all patients were transferred to the ICU, where a forced-air warming blanket was applied unless the patient’s body temperature exceeded 37 °C. Postoperative temperature data from pulmonary artery catheter recording were collected hourly during the first 12 h after ICU admission, with the highest value selected if multiple measurements were available within an hour.

### 2.4. Study Endpoints

The primary study endpoint was severe AKI, defined as Kidney Disease: Improving Global Outcomes (KDIGO) stage 2–3 [14]. The secondary endpoint was non-recovery AKI, defined as stage ≥ 1 persisting for ≥72 h [15,16]. We examined their association with central body temperature trajectories during the first 12 postoperative hours, a period chosen because most temperature changes occurred within this timeframe [6] and it aligns with the typical duration of pulmonary artery catheter monitoring in patients who have undergone cardiac surgery at our institution.

### 2.5. Statistical Analyses

All statistical analyses were performed using SAS version 9.4 (SAS Institute, Cary, NC, USA) and R software (version 4.3.0; https://www.R-project.org (accessed on 20 February 2026)). *p*-values < 0.05 were considered statistically significant.

#### 2.5.1. Temperature Trajectory Modeling

Postoperative 12 h central temperature changes were analyzed using the longitudinal k-means (KML) method in the R package “latrend”. The optimal number of clusters was determined using the Bayesian Information Criterion (BIC). Trajectory analysis is an unbiased method that classifies longitudinal data into groups based on temporal patterns, identifying latent subpopulations with shared characteristics [17]. All patients had recorded ICU admission temperatures and at least one postoperative measurement, with a maximum of five missing values per individual. Missing values were imputed using the mean of the available temperatures for each patient to preserve the overall trajectory shape while minimizing bias. To assess the robustness of this approach, sensitivity analyses were performed using two alternative imputation methods: last observation carried forward (LOCF) and linear interpolation. Trajectory classifications derived from each method were compared using the agreement rate and Cohen’s kappa coefficient. In addition, patterns of missingness across time points were examined, and baseline clinical characteristics and primary outcome were compared between patients with complete and missing data. In addition, implausible temperature values (<32 °C or >40 °C) were reviewed, but none were identified.

Once temperature trajectories were grouped into classes, patient demographics, clinical data, perioperative laboratory findings, and postoperative outcomes were compared between classes. Non-normally distributed continuous variables were analyzed using the Kruskal–Wallis test and presented as medians (interquartile ranges). Categorical variables were compared using the chi-square test and presented as counts (percentages). Post hoc pairwise comparisons between classes were performed with Bonferroni correction.

#### 2.5.2. Association Between Acute Kidney Injury and Temperature Trajectory Classes

Logistic regression analysis was used to examine the association between temperature trajectory classes and severe AKI and non-recovery AKI. Variables with *p* < 0.05 in univariable analysis were included in the multivariable analysis, adhering to the rule of 10 to enhance the predictive power of the model. To prevent multicollinearity, we excluded variables included in EuroSCORE II, such as age, sex, diabetes, chronic kidney disease, chronic lung disease, recent myocardial infarction, congestive heart failure, left ventricular ejection fraction, operation type, and emergency status. We also excluded preoperative hemoglobin and platelet count considering their association with the need for intraoperative transfusion. There was no multicollinearity between the remaining variables. Multivariable logistic regression analysis was performed using an enter method, and the odds ratio (OR) and corresponding 95% confidence interval (CI) were calculated for each variable.

#### 2.5.3. Stastical Methods for Sensitivity and Subgroup Analysis

For the sensitivity analysis, we calculated the E-value to estimate the minimum degree of strength that an unmeasured confounder would need to have with both the exposure and outcome to fully explain the observed association [18] based on the logistic regression results. Given that EuroSCORE II was not specifically developed for AKI prediction, we constructed an alternative multivariable model in which the composite EuroSCORE II was replaced with its individual components (age, sex, diabetes mellitus, chronic kidney disease, myocardial infarction within 3 months, chronic lung disease, left ventricular ejection fraction, and emergency status). Additionally, to address temporal precedence between exposure and outcome, we repeated the analysis after excluding patients who developed severe AKI within the first 12 postoperative hours. A predefined subgroup analysis was also performed in patients with pre-existing chronic kidney disease (eGFR < 60 mL/min/1.73 m^2^).

## 3. Results

**Figure 1:**
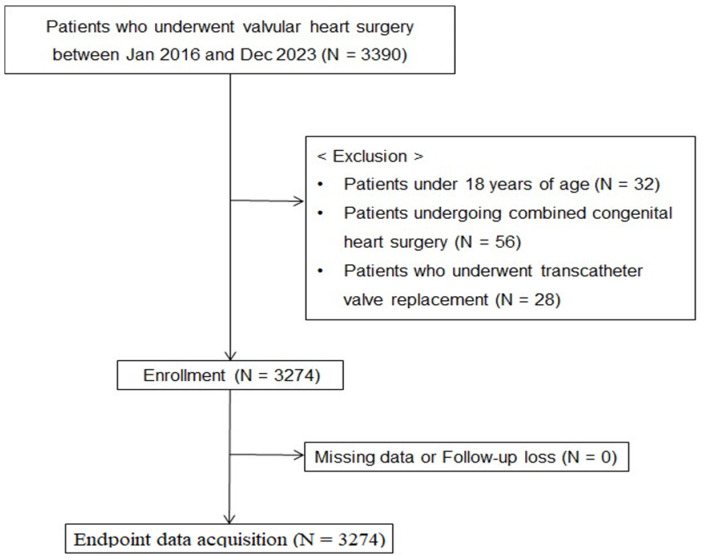
Study flowchart.

From the initial cohort of 3390 patients, we excluded 32 who were <18 years of age, 56 who underwent combined congenital heart surgery, and 28 who underwent transcatheter valve replacement (Figure 1).

### 3.1. Temperature Trajectory Classes and Baseline Characteristics

Of the 3274 patients included in this study, complete 12 h temperature records were available for 2487 (76.0%) of them. Among those with missing values, most had only a single missing observation (*n* = 630, 80.0%), and missing values were sporadically distributed across time points without temporal clustering (Supplementary Figure S1). Baseline characteristics and outcome were largely comparable between patients with complete and incomplete data, although EuroSCORE II was higher and the proportion of females was greater among patients with incomplete data (Supplementary Table S1). Trajectory classifications were highly consistent across the three imputation methods (mean imputation, LOCF, and linear interpolation), with agreement rates of 97.95% (κ = 0.972) and 98.96% (κ = 0.986) for LOCF and linear interpolation compared with mean imputation, respectively (Supplementary Table S3, Figure S2).

**Figure 2:**
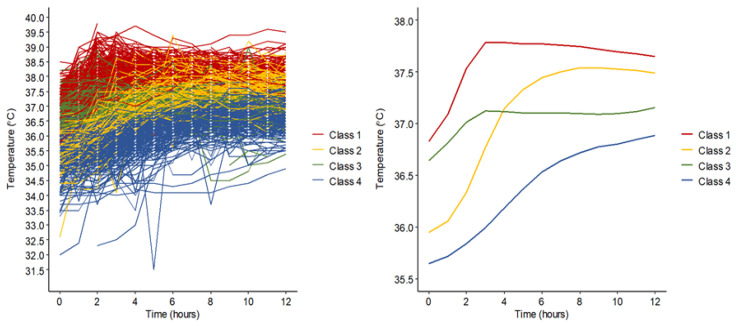
Temperature trajectory classes based on 12 h postoperative central temperature changes.

Four distinct postoperative temperature trajectory classes were identified: Class 1, initial normothermia (36.5–37.5 °C) progressing to mild hyperthermia (37.5–38.0 °C) (32.8% of patients); Class 2, initial mild hypothermia (36.0–36.5 °C) resolving to normothermia (27.4%); Class 3, stable normothermia (24.4%); and Class 4, initial lower-range mild hypothermia (35.5–36.0 °C) with delayed recovery to normothermia (15.4%) (Figure 2). At ICU admission, the patients’ temperatures differed across classes. In Class 1, patient temperature started at 36.8 °C (36.5–37.1 °C), rose to 37.5 °C within 2 h, and remained stable. In Class 2, it started at 36.0 °C (35.7–36.2 °C) and reached normothermia within 2 h. In Class 3, it began at 36.6 °C (36.4–36.9 °C) and remained stable. In Class 4, it started at 35.7 °C (35.3–36.1 °C) and required 5 h to reach normothermia. After stabilization, patient temperature in Classes 1–3 remained ≥ 37.0 °C, whereas that in Class 4 stayed below 37.0 °C (Supplementary Figure S3).

Comparisons of patient demographics, clinical data, and preoperative laboratory results between trajectory classes are shown in Table 1 and Supplementary Table S2. The patients in Classes 1–3 had similar characteristics, whereas those in Class 4 were older and had a higher prevalence of chronic kidney disease, higher EuroSCORE II, and greater prevalence of emergency surgery compared with the patients in Classes 1–3. The proportion of patients receiving intraoperative or postoperative packed erythrocyte transfusions and the number of units received per patient were significantly higher in Class 4 compared with other classes. Preoperative hemoglobin, platelet count, eGFR, and albumin levels were lower, while creatinine and C-reactive protein levels were higher in Class 4 patients.

### 3.2. Severe AKI and Non-Recovery AKI Among Temperature Trajectory Classes

Severe AKI (KDIGO stages 2–3) occurred in 180 patients (5.5%), with those in Class 4 showing the highest incidence and severity of AKI. The rate of severe AKI was significantly higher in Class 4 patients (15.1%) than in those in Classes 1 (2.9%), 2 (3.9%), and 3 (4.8%) (*p* < 0.001 for all comparisons). Non-recovery AKI was also most frequent in Class 4 patients (15.1%), followed by those in Classes 3 (4.4%), 2 (4.0%), and 1 (1.7%), with rates significantly higher in Class 4 patients than those in the other classes (*p* < 0.001 for all comparisons). Class 4 was also associated with higher rates of prolonged mechanical ventilation (>48 h) and delirium, longer ICU and hospital stays, and higher in-hospital and 30-day mortality compared with the other classes (Table 2).

### 3.3. Risk Factors for Severe AKI and Non-Recovery AKI

In multivariable logistic regression analysis, the independent risk factors for severe AKI were temperature trajectory class, cerebrovascular disease, eGFR, cardiopulmonary bypass time, and intraoperative erythrocyte transfusion. Analyses were conducted using two models. In model 1, the risk of severe AKI was assessed for each trajectory class. After adjusting for selected variables, the ORs for developing severe AKI, compared with Class 4, were as follows: Class 1, 0.39 (95% CI: 0.24–0.65, *p* < 0.001); Class 2, 0.36 (95% CI: 0.22–0.59, *p* < 0.001); and Class 3, 0.47 (95% CI: 0.30–0.74, *p* = 0.001). In model 2, Classes 1, 2, and 3 were combined, and the adjusted OR for the combined group was 0.41 (95% CI: 0.28–0.59, *p* < 0.001) (Table 3).

The same variables were identified as independent risk factors for non-recovery AKI. After adjustment, the ORs for non-recovery AKI were significantly lower in Classes 1, 2, and 3 compared with those in Class 4. The adjusted OR for non-recovery AKI was also lower in the combined group (Classes 1 + 2 + 3) than in Class 4 (*p* < 0.001) (Table 4).

### 3.4. Sensitivity and Subgroup Analysis

In the primary multivariable model adjusted for EuroSCORE II, the E-values for the ORs of severe AKI in Classes 1, 2, and 3 (vs. Class 4) were 4.51, 4.97, and 3.70, respectively. For non-recovery AKI, the corresponding E-values were 7.98, 4.49, and 4.09. These results indicate that an unmeasured confounder would need a risk ratio ≥3.70 (for severe AKI) or ≥4.09 (for non-recovery AKI) with both exposure and outcome to fully explain the associations (Table 3 and Table 4). In the alternative multivariable model replacing the composite EuroSCORE II with its individual clinical components, temperature trajectory remained independently associated with both severe and non-recovery AKI. Compared with Class 4, the adjusted ORs for severe AKI were 0.43 (95% CI: 0.26–0.71) for Class 1, 0.38 (95% CI: 0.23–0.61) for Class 2, and 0.50 (95% CI: 0.32–0.79) for Class 3 (Supplementary Table S4), with similar findings observed for non-recovery AKI (Supplementary Table S5).

To address temporal precedence, we repeated the analysis after excluding the five patients who developed severe AKI within the first 12 postoperative hours. The Class 4 trajectory remained a significant predictor of severe AKI (using Class 4 as the reference: Class 1, OR 0.41, 95% CI: 0.25–0.67; Class 2, OR 0.37, 95% CI: 0.23–0.60; Class 3, OR 0.46, 95% CI: 0.29–0.72; all *p* < 0.001; Supplementary Table S6).

In a subgroup analysis of patients with pre-existing chronic kidney disease (eGFR < 60 mL/min/1.73 m^2^, *n* = 498), patients in Class 4 had significantly higher rates of both severe and non-recovery AKI compared with those in other classes. The association between Class 4 and these outcomes was stronger than in the overall cohort (combined group OR 0.31, 95% CI: 0.18–0.55, *p* < 0.001; Supplementary Table S7).

### 3.5. Predictors of Temperature Trajectory in Class 4

In the multivariable logistic regression analysis, EuroSCORE II (OR 1.04, 95% CI: 1.01–1.06, *p* = 0.003), eGFR (OR 0.98, 95% CI: 0.976–0.984, *p* < 0.001), and intraoperative erythrocyte transfusion (OR 1.28, 95% CI: 1.17–1.40, *p* < 0.001) were identified as independent predictors of Class 4 (Supplementary Table S8).

## 4. Discussion

In this retrospective cohort study, we identified four distinct temperature trajectory classes during the first 12 postoperative hours and assessed their associations with severe AKI. A trajectory characterized by initial lower-range mild hypothermia with delayed recovery to normothermia was associated with increased risks of severe AKI and non-recovery AKI. However, this pattern may reflect greater perioperative illness severity and physiological instability rather than a direct causal relationship with subsequent renal dysfunction.

Cardiac surgery-associated AKI is a well-recognized complication, and even transient or mild AKI substantially increases the risks of mortality and progression to chronic kidney disease [19,20], with severe or persistent AKI associated with a three- to eightfold higher risk of death [9,11]. Given its clinical impact and limited treatment options, identifying modifiable risk factors for severe AKI is crucial. In this context, perioperative temperature abnormalities are common and have been associated with poor postoperative outcomes [5,6], yet their effect on renal function remains unclear. Hypothermia is often considered a maladaptive response, reflecting more serious conditions such as inflammation, thermodysregulation, and multi-organ failure [21,22], whereas hyperthermia may represent an adaptive response that supports immune function and pathogen elimination [23]. In relation to renal function, hypothermia has been associated with AKI in non-cardiac surgeries [24,25], while hyperthermia has also been linked to AKI following cardiac surgery [26,27]. However, evidence on the relationship between temperature abnormalities and severe AKI remains limited, particularly in valvular heart surgery patients, a population with a high incidence of renal dysfunction [19,20]. In addition, prior studies have shown inconsistent findings, partly due to heterogeneity in temperature measurement timing, sites, and data analysis methods. To address these limitations, we applied latrend cluster analysis to classify 12 h postoperative central body temperature trajectories.

We identified four distinct temperature trajectory classes. Rewarming was performed to ensure that the patient’s body temperature was above 36.5 °C during weaning from cardiopulmonary bypass; however, patient temperature fell below 36.0 °C at ICU admission in 49% of patients in Classes 2 and 4. Class 4, characterized by initial lower-range mild hypothermia and a delayed 5 h recovery to normothermia, exhibited significantly higher rates and risks of severe and non-recovery AKI. In contrast, Class 2, with initial mild hypothermia that resolved within 2 h, showed risks similar to those demonstrated by Classes 1 and 3, which maintained normothermia. The absolute differences in initial temperatures between classes were modest (approximately 0.5–1.0 °C), suggesting that a purely thermally mediated mechanism may not fully account for the observed associations with AKI outcomes. Consistent with this interpretation, patients in Class 4 had a higher burden of preoperative comorbidities affecting thermoregulation and received greater volumes of intraoperative erythrocyte transfusions. These trajectory patterns may therefore reflect overall perioperative physiological stress, including impaired thermoregulation, inflammation, hemodynamic instability, and transfusion-related factors, all of which have been associated with AKI risk. In this context, Class 4 likely represents a clinically observable high-risk phenotype rather than a direct mechanistic driver of renal injury. Accordingly, temperature trajectory may serve as an integrated indicator of global physiological stress rather than an independent determinant of AKI. Notably, the association that persisted after adjusting for clinical covariates, including pre-existing renal dysfunction, was more pronounced among patients with pre-existing chronic kidney disease (eGFR < 60 mL/min/1.73 m^2^). All E-values for the observed ORs were ≥3.7, indicating that unmeasured confounders would need to have a substantial effect on severe and non-recovery AKI to negate our findings. Additional sensitivity analyses—including an alternative model replacing the composite EuroSCORE II with its individual components and exclusion of patients who developed severe AKI within the first 12 postoperative hours—produced consistent results. Collectively, these findings support the potential utility of early postoperative temperature trajectories for risk stratification, although causality cannot be inferred from this observational analysis.

Several potential pathophysiological mechanisms may link postoperative hypothermia to the development of AKI. While metabolic suppression under hypothermia can theoretically reduce oxygen demand, its detrimental effects in the postoperative period may predominate. Hypothermia induces a leftward shift in the oxyhemoglobin dissociation curve, which increases hemoglobin’s affinity for oxygen and subsequently impairs oxygen delivery to the renal medulla, an area already vulnerable to hypoxia [28]. Furthermore, hypothermia-induced systemic vasoconstriction and increased blood viscosity can further reduce renal blood flow and impair microcirculatory perfusion [29]. These factors, combined with the potential for hypothermia to exacerbate coagulopathy and inflammatory responses, may create a milieu that predisposes the kidneys to ischemic injury. However, as noted, these physiological changes may also occur in parallel with other indicators of severe illness, making it difficult to isolate hypothermia as the sole causal driver. Importantly, previous studies demonstrating the beneficial effects of hypothermia on renal function in cardiac surgery were conducted during the rewarming phase of cardiopulmonary bypass [26,27,30], where it may have mitigated renal ischemia–reperfusion injury. In contrast, in our study, temperature differences between classes were minimal at bypass weaning but became more evident at ICU admission and within hours thereafter, during which the detrimental effects of hypothermia emerged. Postoperative hypothermia has been associated with reduced renal function and an increased incidence of AKI after major abdominal surgery [24], consistent with our findings. Notably, Class 4, characterized by delayed recovery to normothermia, exhibited worse outcomes, including prolonged mechanical ventilation, delirium, longer ICU and hospital stays, and higher short-term mortality, whereas Class 2, with rapid recovery to normothermia, did not. This aligns with a previous observation that patients initially hypothermic after surgery but later reaching normothermia had lower mortality than those who remained hypothermic [6]. These findings highlight the clinical relevance of early postoperative temperature changes from cardiopulmonary bypass weaning through the immediate postoperative period. The identified patterns were associated with an increased risk of severe AKI and may support early risk stratification after cardiac surgery.

The main strength of this study is that it provides primary evidence on the potential utility of early postoperative temperature trajectories as a prognostic marker for key clinical outcomes in a relatively large cohort of cardiac surgery patients. We conducted trajectory-based modeling with complementary sensitivity analyses to enhance the robustness of our findings. However, several limitations of this study should be considered. Firstly, although postoperative hypothermia was associated with severe AKI, the observational design of this study precludes causal inference. It remains uncertain whether hypothermia directly contributes to renal dysfunction or merely reflects underlying clinical instability, disease severity, or unmeasured hemodynamic factors. Therefore, our findings should be interpreted as identifying a high-risk phenotype that reflects a patient’s overall physiological vulnerability rather than establishing a direct biological pathway. Further investigations into the mechanisms linking hypothermia to renal injury are required. Accordingly, prospective studies, including randomized or protocolized temperature management trials, are needed to determine whether targeted temperature interventions can modify AKI risk before clinical recommendations are warranted. Secondly, we did not have access to detailed intraoperative hypotension exposure metrics (e.g., duration or area under threshold below specific mean arterial pressure values) or standardized definitions of postoperative shock. These variables represent major unmeasured confounders, as hemodynamic instability is a well-established determinant of renal injury and may influence both postoperative temperature trajectories and AKI risk. Although postoperative vasoactive drug support was available and explored as a crude surrogate of hemodynamic compromise, this variable lacks the granularity required to capture hypotension burden or shock severity. Therefore, residual confounding related to unmeasured hemodynamic parameters may have influenced our findings. Future prospective studies incorporating high-resolution intraoperative hemodynamic monitoring and standardized shock metrics are warranted to better delineate these relationships. Thirdly, AKI was defined solely based on serum creatinine criteria, without incorporating urine output. Because serum creatinine typically rises 24–48 h after kidney injury, the exclusion of urine output may have reduced sensitivity for detecting early or transient AKI, potentially leading to underestimation or misclassification of AKI events [31]. Such non-differential misclassification of the outcome would likely bias the observed associations toward the null, suggesting that the observed effects may have been underestimated. However, urine output is also influenced by perioperative fluid management, diuretic use, and hemodynamic factors, and its inclusion could introduce additional variability. Therefore, the direction and magnitude of the association might differ if the full KDIGO criteria were applied. This limitation should be considered when interpreting these findings. Fourthly, this retrospective study was subject to inherent data omissions, including missing temperature measurements. Although the overall rate of missing values was low and most were isolated single-hour omissions, patients with missing data had higher baseline clinical severity (median EuroSCORE II 4.0 vs. 2.0, *p* < 0.001). However, the incidence of severe AKI was comparable between patients with complete and incomplete data (5.7% vs. 5.4%, *p* = 0.756; Supplementary Table S1). To evaluate whether our imputation strategy introduced systematic bias, we performed additional sensitivity analyses using last observation carried forward and linear interpolation. These analyses yielded results that were almost identical to those of the primary analysis (agreement > 97%; Cohen’s kappa > 0.97), indicating that the findings were robust across imputation methods and unlikely to be driven by bias related to data completeness. Finally, while body temperature trajectories provide valuable insights for risk prediction, they do not allow for a precise determination of the optimal temperature range to minimize risk.

## 5. Conclusions

In conclusion, we identified four distinct postoperative temperature trajectories over the first 12 h. A trajectory characterized by initial lower-range mild hypothermia with delayed recovery to normothermia was associated with increased risks of both severe and non-recovery AKI. These findings suggest that early postoperative temperature patterns may serve as a clinical marker of AKI risk and inform perioperative risk stratification from cardiopulmonary bypass weaning through the early postoperative period. Prospective studies are needed to determine whether targeted temperature management can modify the risk of AKI after cardiac surgery.

## Figures and Tables

**Table 1:** Demographic, clinical, and perioperative transfusion data.

	All (*n* = 3274)	Class 1 (*n* = 1075, 32.8%)	Class 2 (*n* = 897, 27.4%)	Class 3 (*n* = 798, 24.4%)	Class 4 (*n* = 504, 15.4%)	*p*-Value
Age (years)	65 (56, 72)	61 (51, 68) *	65 (57, 73) *	66 (57, 73) *	71 (63, 76)	<0.001
Female, n (%)	1630 (49.8%)	501(46.6%) *	441 (49.2%)	413 (51.8%)	275 (54.6%)	0.016
Body mass index (kg/m^2^)	23.6 (21.5, 25.9)	24.1 (22.0, 26.3) *	23.3 (21.0, 25.6)	23.5 (21.5, 26.1) *	23.1 (20.8, 25.1)	<0.001
Morbidity, n (%)						
Hypertension	1638 (50.0%)	494 (46.0%) *	459 (51.2%)	407 (51.0%)	278 (55.2%)	0.005
Diabetes mellitus	637 (19.5%)	201 (18.7%)	153 (17.1%)	170 (21.3%)	113 (22.4%)	0.041
Chronic kidney disease	302 (9.2%)	46 (4.3%) *	74 (8.2%) *	78 (9.8%) *	104 (20.6%)	<0.001
MI within 3 months	99 (3.0%)	31 (2.9%)	27 (3.0%)	29 (3.6%)	12 (2.4%)	0.616
CVD	442 (13.5%)	128 (11.9%)	105 (11.7%)	125 (15.7%)	84 (16.7%)	0.006
Chronic lung disease	122 (3.7%)	28 (2.6%) *	35 (3.9%)	28 (3.5%)	31 (6.2%)	0.007
CHF	720 (22.0%)	155(14.4%) *	183 (20.4%) *	225 (28.2%)	157 (31.2%)	<0.001
EuroSCORE II	2.5 (1.1, 5.0)	2.0 (1.0, 4.0) *	2.7(1.1, 5.0) *	2.9(1.3, 6.0) *	3.1(1.4, 7.3)	<0.001
LVEF (%)	64 (57, 70)	65 (59, 71) *	64 (57, 70)	63 (55, 70)	64 (56, 70)	0.001
Medication						
Beta blocker	1136 (34.7%)	360 (33.5%)	278 (31.0%) *	306 (38.3%)	192 (38.1%)	0.004
Calcium channel blocker	831 (25.4%)	221(20.6%) *	231 (25.8%) *	214 (26.8%)	165 (32.7%)	<0.001
RASi	1645 (50.2%)	513 (47.7%)	451 (50.3%)	418 (52.4%)	263 (52.2%)	0.175
Statin	1467 (44.8%)	451 (42.0%)	406 (45.3%)	373 (46.7%)	237 (47.0%)	0.120
Diuretics	2184 (66.7%)	662(61.6%) *	568 (63.3%) *	586 (73.4%)	368 (73.0%)	<0.001
Type of surgery						
Aortic valve surgery	961 (29.4%)	304 (28.3%)	291 (32.4%)	213 (26.7%)	153 (30.4%)	<0.001
Mitral valve surgery	1175 (35.9%)	399 (37.1%)	266 (29.7%)	333 (41.7%)	177 (35.1%)	
Double valve surgery	421 (12.9%)	139 (12.9%)	97 (10.8%)	124 (15.5%)	61 (12.1%)	
Valve + CABG surgery	204 (6.2%)	72 (6.7%)	58 (6.5%)	51 (6.4%)	23 (4.6%)	
Valve + aorta surgery	313 (9.6%)	105 (9.8%)	116 (12.9%)	46 (5.8%)	46 (9.1%)	
Tricuspid valve surgery	200 (6.1%)	56 (5.2%)	69 (7.7%)	31 (3.9%)	44 (8.7%)	
Emergency operation	40 (1.2%)	10 (0.9%) *	8 (0.9%) *	7 (0.9%) *	15 (3.0%)	0.002
CPB time (min)	95 (70, 127)	97 (71, 125)	92 (66, 130)	95 (70, 124)	94 (67, 131)	0.362
ACC time (min)	65 (45, 90)	68 (47, 90)	65 (43, 90)	65 (46, 88)	63 (44, 92)	0.196
Perioperative erythrocyte transfusion						
Intraoperative transfusion (n)	1212 (37.0%)	320 (29.8%) *	337 (37.6%) *	304 (38.1%) *	251(49.8%)	<0.001
Intraoperative transfusion (unit)	0 (0, 1)	0 (0, 1) *	0 (0, 1) *	0 (0, 1) *	0 (0, 2)	<0.001
Postoperative transfusion (n)	1453 (44.5%)	383 (35.7%) *	406 (45.4%) *	361 (45.4%) *	303 (60.2%)	<0.001
Postoperative transfusion (unit)	0 (0, 2)	0 (0, 1) *	0 (0, 2) *	0 (0, 2) *	1 (0, 4)	<0.001

Values are median (interquartile range) or number (percent). MI, myocardial infarction; CVD, cerebrovascular disease; CHF, congestive heart failure; EuroSCORE, European System for Cardiac Operative Risk Evaluation; LVEF, left ventricular ejection fraction; RASi, renin–angiotensin system inhibitor; CABG, coronary artery bypass graft; CPB, cardiopulmonary bypass; ACC, aorta cross-clamp; *, *p* < 0.05 compared with Class 4.

**Table 2:** Postoperative outcomes during hospitalization.

	All (*n* = 3274)	Class 1 (*n* = 1075, 32.8%)	Class 2 (*n* = 897, 27.4%)	Class 3 (*n* = 798, 24.4%)	Class 4 (*n* = 504, 15.4%)	*p*-Value
Severe AKI	180 (5.5%)	31 (2.9%) *	35 (3.9%) *	38 (4.8%) *	76 (15.1%)	<0.001
Non-recovery AKI	165 (5.0%)	18 (1.7%) *	36 (4.0%) *	35 (4.4%) *	76 (15.1%)	<0.001
AKI occurrence	722 (22.1%)	196 (18.2%) *	184 (20.5%) *	180 (22.6%) *	162 (32.1%)	<0.001
AKI severity		*	*	*		
Non-AKI	2552 (77.9%)	879 (81.8%)	713 (79.5%)	618 (77.4%)	342 (67.9%)	<0.001
Stage 1	542 (16.6%)	165 (15.3%)	149 (16.6%)	142 (17.8%)	86 (17.1%)	
Stage 2	67 (2.0%)	24 (2.2%)	15 (1.7%)	20 (2.5%)	8 (1.6%)	
Stage 3	113 (3.5%)	7 (0.7%)	20 (2.2%)	18 (2.3%)	68 (13.5%)	
Reoperation due to bleeding	168 (5.1%)	31 (2.9%) *	45 (5.0%) *	47 (5.9%)	45 (8.9%)	<0.001
Mechanical ventilation > 48 h	125 (3.8%)	13 (1.2%) *	34 (3.8%) *	24 (3.1%) *	53 (10.5%)	<0.001
Myocardial infarction	4 (0.1%)	0	3 (0.3%)	1 (0.1%)	0	0.154
Wound infection	58 (1.8%)	19 (1.8%)	20 (2.2%)	11 (1.4%)	8 (1.6%)	0.594
Stroke	80 (2.4%)	28 (2.6%)	19 (2.1%)	16 (2.0%)	17 (3.4%)	0.395
Delirium	188 (5.7%)	33 (3.1%) *	38 (4.2%) *	51 (6.4%) *	66 (13.1%)	<0.001
In-hospital mortality	53 (1.6%)	8 (0.7%) *	11 (1.2%) *	11 (1.4%) *	23 (4.6%)	<0.001
30-day mortality	28 (0.9%)	3 (0.3%) *	6 (0.7%) *	6 (0.8%) *	13 (2.6%)	<0.001
Intensive care unit stay (day)	3 (2, 3)	3 (2, 3) *	3 (2, 3) *	3 (2, 4) *	3 (2, 4)	<0.001
Hospital stay (day)	11 (9, 15)	10 (9, 14) *	10 (9, 15) *	11 (9, 15) *	12 (9, 20)	<0.001

Values are number (percent) or median (interquartile range). Severe AKI, KDIGO stage 2–3; non-recovery AKI, KDIGO stage ≥ 1 lasting ≥ 72 h; *, *p* < 0.05 compared with Class 4.

**Table 3:** Logistic regression analysis of risk factors for severe acute kidney injury (primary model adjusted for EuroSCORE II).

	Univariable Analysis		Multivariable Analysis (1)			Multivariable Analysis (2)		
Variables	OR (95% CI)	*p*-Value	OR (95% CI)	*p*-Value	E-Value	OR (95% CI)	*p*-Value	E-Value
Temperature trajectory								
Class 4	reference		reference			reference		
Class 1	0.17 (0.11, 0.26)	<0.001	0.39 (0.24, 0.65)	<0.001	4.51	-	-	
Class 2	0.23 (0.15, 0.35)	<0.001	0.36 (0.22, 0.59)	<0.001	4.97	-	-	
Class 3	0.28 (0.19, 0.42)	<0.001	0.47 (0.30, 0.74)	0.001	3.70	-	-	
Class 1 + 2 + 3	0.22 (0.16, 0.30)	<0.001	-	-		0.41 (0.28, 0.59)	<0.001	4.33
Cerebrovascular disease	2.19 (1.54, 3.13)	<0.001	1.66 (1.10, 2.49)	0.015	2.70	1.68 (1.12, 2.51)	0.013	2.74
EuroSCORE II	1.12(1.09, 1.15)	<0.001	1.02 (0.99, 1.06)	0.150	1.18	1.03 (0.99, 1.06)	0.126	1.19
CPB time (min)	1.01 (1.01, 1.01)	<0.001	1.01 (1.00, 1.01)	<0.001	1.09	1.01 (1.00, 1.01)	<0.001	1.09
Transfusion *	1.68 (1.52, 1.85)	<0.001	1.25 (1.10, 1.42)	<0.001	1.81	1.25 (1.10, 1.41)	0.001	1.80
eGFR (mL/min/1.73 m^2^)	0.97 (0.96, 0.97)	<0.001	0.97 (0.97, 0.98)	<0.001	1.20	0.97 (0.97, 0.98)	<0.001	1.20
Vasoactive drug	1.12 (0.27, 4.68)	0.876						

EuroSCORE, European System for Cardiac Operative Risk Evaluation; CPB, cardiopulmonary bypass; eGFR, estimated glomerular filtration rate; OR, odds ratio; CI, confidence interval; *, intraoperative erythrocyte transfusion unit.

**Table 4:** Logistic regression analysis of risk factors for non-recovery AKI (primary model adjusted for EuroSCORE II).

	Univariable Analysis		Multivariable Analysis (1)			Multivariable Analysis (2)		
Variables	OR (95% CI)	*p*-Value	OR (95% CI)	*p*-Value	E-Value	OR (95% CI)	*p*-Value	E-Value
Temperature trajectory								
Class 4	reference		reference			reference		
Class 1	0.10 (0.06, 0.16)	<0.001	0.24 (0.13, 0.42)	<0.001	7.98	-	-	
Class 2	0.24 (0.16, 0.36)	<0.001	0.40 (0.25, 0.64)	<0.001	4.49	-	-	
Class 3	0.26 (0.17, 0.39)	<0.001	0.43 (0.27, 0.68)	<0.001	4.09	-	-	
Class 1 + 2 + 3	0.19 (0.14, 0.26)	<0.001	-	-		0.36 (0.25, 0.53)	<0.001	4.97
Cerebrovascular disease	2.48 (1.73, 3.55)	<0.001	1.87 (1.23, 2.83)	0.003	3.14	1.88 (1.24, 2.84)	0.003	3.16
EuroSCORE II	1.12 (1.09, 1.15)	<0.001	1.01 (0.98, 1.04)	0.634	1.10	1.01 (0.98, 1.05)	0.538	1.11
CPB time(min)	1.01 (1.01, 1.01)	<0.001	1.01 (1.01, 1.01)	<0.001	1.10	1.01 (1.01, 1.01)	<0.001	1.10
Transfusion *	1.69 (1.54, 1.87)	<0.001	1.28 (1.13, 1.46)	<0.001	1.88	1.29 (1.13, 1.47)	<0.001	1.90
eGFR (mL/min/1.73 m^2^)	0.96 (0.95, 0.97)	<0.001	0.97 (0.96, 0.98)	<0.001	1.22	0.97 (0.96, 0.98)	<0.001	1.22
Vasoactive drug	2.13 (0.29, 15.62)	0.456						

EuroSCORE, European System for Cardiac Operative Risk Evaluation; CPB, cardiopulmonary bypass; eGFR, estimated glomerular filtration rate; OR, odds ratio; CI, confidence interval; *, intraoperative erythrocyte transfusion unit.

## Data Availability

Data supporting the findings of this study are available from the corresponding author upon reasonable request.

## Supplementary Materials

The following supporting information can be downloaded at the following link: https://www.mdpi.com/article/10.3390/jcm15051887/s1. Table S1: Comparison of baseline and outcomes by data completeness. Table S2: Preoperative laboratory findings among temperature trajectory classes. Table S3: Sensitivity analysis of trajectory classification across different imputation methods. Table S4: Logistic regression analysis of risk factors for severe AKI adjusted for individual EuroSCORE II components (alternative model). Table S5: Logistic regression analysis of risk factors for non-recovery AKI adjusted for individual EuroSCORE II components (alternative model). Table S6: Logistic regression analysis of risk factors for severe AKI excluding patients diagnosed within the first 12 postoperative hours. Table S7: Logistic regression analysis of risk factors for severe AKI in patients with preexisting chronic kidney disease (eGFR < 60 mL/min/1.73 m^2^). Table S8: Logistic regression analysis of risk factors for Class 4 temperature trajectory. Figure S1: Visulaization of missing data patterns. Figure S2: Comparison of three different imputation methods for temperature data over time. Figure S3: Perioperative temperature changes among trajectory classes.

## Abbreviations

The following abbreviations are used in this manuscript:

ACC	aortic cross-clamp
AKI	acute kidney injury
CABG	coronary artery bypass graft
CVD	cerebrovascular disease
CHF	congestive heart failure
eGFR	estimated glomerular filtration rate
ICU	intensive care unit
KDIGO	Kidney Disease Improving Global Outcomes
LVEF	left ventricular ejection fraction
RASi	renin–angiotensin system inhibitor
