# Supplementary material for: The Association Between Early Postoperative Temperature Trajectories and Severe Acute Kidney Injury After Valvular Heart Surgery: A Retrospective Cohort Study
Source: J Clin Med. 2026 Mar 1;15(5):1887. doi: 10.3390/jcm15051887 (PMC12986021; doi:10.3390/jcm15051887)
Supplement: Supplementary file 1 [file jcm-15-01887-s001.zip › jcm-4140551-supplementary.pdf]

Supplementary Table S1. Comparison of baseline and outcomes by data completeness

	Complete data (*n* = 2487)	Missing data (*n*=787)	P value
age (years)	65.0 (56.0, 72.0)	64.0 (55.0, 72.0)	0.162
female, n (%)	1200 (48.3)	430 (54.6)	0.002
Morbidity, n (%)			
hypertension	1249 (50.2)	389 (49.4)	0.691
diabetes mellitus	490 (19.7)	147 (18.7)	0.527
chronic kidney disease	238 (9.6)	64 (8.1)	0.225
MI within 3 months	68 (2.7)	31 (3.9)	0.085
CVD	329 (13.2)	113 (14.4)	0.419
chronic lung disease	89 (3.6)	33 (4.2)	0.428
LVEF (%)	64.0 (57.0, 70.0)	65.0 (57.0, 70.0)	0.707
EuroSCORE II	2.03 (1.04, 4.97)	4.00 [1.90, 7.00]	< 0.001
Severe AKI	135 (5.4)	45 (5.7)	0.756

Values are median (interquartile range) or number (percent). MI, myocardial infarction; CVD, cerebrovascular disease; EuroSCORE, European System for Cardiac Operative Risk Evaluation; LVEF, left ventricular ejection fraction; AKI, acute kidney injury

Supplementary Table S2. Preoperative laboratory findings among trajectory classes

	All (n = 3274)	Class 1 (n = 1075, 32.8%)	Class 2 (n= 897, 27.4%)	Class 3 (n = 798, 24.4%)	Class 4 (n = 504, 15.4%)	p value
hemoglobin (g/dl)	12.7 (11.3-13.9)	13.2 (12.0-14.2)*	12.7 (11.3-13.9)*	12.5(11.2-13.8)*	11.8 (9.9-13.2)	< 0.001
platelet (10^3/μl)	193 (156-237)	202 (166-240)*	191 (155-237)*	190 (153-237)*	179 (141-228)	< 0.001
creatinine (mg/dl)	0.83(0.69-0.99)	0.79 (0.67-0.93)*	0.83 (0.69-0.97)*	0.84 (0.70-1.01)*	0.91 (0.75-1.18)	< 0.001
eGFR (ml/min/1.73m^2)	82.6 (68.0-98.6)	88.2 (74.5-104.0)*	83.5 (69.5-97.7)*	80.6 (66.7-96.7)*	70.6 (53.8-86.6)	< 0.001
glucose (mg/dl)	108 (95-132)	109 (95-132)	107 (94-129)	109 (95-135)	107 (93-139)	0.232
albumin (g/dl)	4.1 (3.8-4.4)	4.2 (3.9-4.5)*	4.1 (3.8-4.4)*	4.1 (3.7-4.4)*	4.0 (3.5-4.3)	< 0.001
C-reactive protein (mg/l)	1.7 (0.6-6.2)	1.4 (0.6-5.2)*	1.3 (0.5-4.7)*	2.2 (0.8-7.7)	2.0 (0.7-9.3)*	< 0.001

Values are median (IQR). eGFR, estimated glomerular filtration rate; *, $p < 0.05$ compared to Class 4

Supplementary Table S3. Sensitivity analysis of trajectory classification across different imputation methods

(A) Mean imputation vs. Last Observation Carried Forward (LOCF)

	LOCF Class 1	LOCF Class 2	LOCF Class 3	LOCF Class 4	Total
Mean class 1	**1059**	3	13	0	1075
Mean class 2	3	**874**	13	7	897
Mean class 3	4	6	**772**	16	798
Mean class 4	0	0	2	**502**	504
Total	1066	883	800	525	3274
Agreement rate: 97.95%, Cohen's kappa : 0.972					

(B) Mean imputation vs. Linear interpolation

	Linear Class 1	Linear Class 2	Linear Class 3	Linear Class 4	Total
Mean class 1	**1074**	0	1	0	1075
Mean class 2	6	**886**	4	1	897
Mean class 3	10	5	**783**	0	798

Mean class 4	0	2	5	**497**	504
Total	1090	893	793	498	3274
Agreement rate: 98.96%, Cohen’s kappa : 0.986					

Agreement rates and Cohen’s kappa coefficients were calculated to assess the robustness of trajectory classification against different missing data imputation strategies.

Supplementary Table S4. Logistic regression analysis of risk factors for severe AKI adjusted for individual EuroSCORE II components (alternative model)

	Univariable analysis		Multivariable analysis (1)			Multivariable analysis (2)		
Variables	OR (95% CI)	*p value*	OR (95% CI)	*p value*	*E value*	OR (95% CI)	*p* value	*E value*
Temperature trajectory								
Class 4	reference		reference			reference		
Class 1	0.17 (0.11, 0.26)	< 0.001	0.43 (0.26, 0.71)	< 0.001	4.13	-	-	
Class 2	0.23 (0.15, 0.35)	< 0.001	0.38 (0.23, 0.61)	< 0.001	4.71	-	-	
Class 3	0.28 (0.19, 0.42)	< 0.001	0.50 (0.32, 0.79)	0.003	3.39	-	-	
Class 1+2+3	0.22 (0.16, 0.30)	< 0.001	-	-		0.44 (0.30, 0.63)	< 0.001	4.01
cerebrovascular disease	2.19 (1.54, 3.13)	< 0.001	1.60 (1.06, 2.40)	0.024	2.57	1.62 (1.08, 2.43)	0.020	2.62
CPB time (min)	1.01 (1.01, 1.01)	< 0.001	1.01 (1.00, 1.01)	< 0.001	1.09	1.01 (1.00, 1.01)	< 0.001	1.09
transfusion*	1.68 (1.52, 1.85)	< 0.001	1.29 (1.14, 1.47)	< 0.001	1.91	1.29 (1.14, 1.46)	0.001	1.89
eGFR (ml/min/1.73m^2)	0.97 (0.96, 0.97)	< 0.001	0.98 (0.98, 0.99)	< 0.001	1.14	0.98 (0.98, 0.99)	< 0.001	1.14
age (years)	1.03 (1.02, 1.05)	<0.001	1.00 (0.99, 1.02)	0.713	1.06	1.00 (0.99, 1.02)	0.694	1.06
female, n (%)	0.99 (0.73, 1.33)	0.925	0.82 (0.57, 1.17)	0.279	1.74	0.82 (0.58, 1.18)	0.293	1.72
diabetes mellitus, n (%)	2.30 (1.67, 3.17)	<0.001	1.60 (1.10, 2.32)	0.014	2.57	1.59 (1.09, 2.31)	0.016	2.55
CKD, n (%)	6.62 (4.75, 9.23)	<0.001	2.70 (1.72, 4.24)	<0.001	4.85	2.69 (1.71, 4.22)	<0.001	4.82
MI wihtin 3 months, n (%)	1.99 (1.02, 3.89)	0.045	0.93 (0.42, 2.06)	0.850	1.38	0.93 (0.42, 2.07)	0.856	1.37
CLD, n (%)	2.13 (1.18, 3.87)	0.013	1.40 (0.72, 2.71)	0.325	2.14	1.39 (0.72, 2.70)	0.328	2.13
CHF, n (%)	2.39 (1.75, 3.27)	<0.001	1.57 (1.08, 2.28)	0.019	2.51	1.59 (1.09, 2.31)	0.015	2.56
LVEF (%)	0.99 (0.98, 1.00)	0.029	1.01 (0.99, 1.02)	0.327	1.09	1.01 (0.99, 1.02)	0.339	1.09
Emergency surgery, n (%)	6.89 (3.38, 14.02)	<0.001	4.07 (1.62, 10.23)	0.003	7.61	4.09 (1.63, 10.27)	0.003	7.64

CPB, cardiopulmonary bypass; eGFR, estimated glomerular filtration rate; CKR, chronic kidney disease; MI, myocardial infarction; CLD, chronic lung disease; CHF, congestive heart failure; LEVF, left ventricular ejection fraction; OR, odds ratio; CI, confidence interval; *, intraoperative erythrocyte transfusion unit

Supplementary Table S5. Logistic regression analysis of risk factors for non-recovery AKI adjusted for individual EuroSCORE II components (alternative model)

	Univariable analysis		Multivariable analysis (1)			Multivariable analysis (2)		
Variables	OR (95% CI)	*p value*	OR (95% CI)	*p value*	*E value*	OR (95% CI)	*p* value	*E value*
Temperature trajectory								
Class 4	reference		reference			reference		
Class 1	0.10 (0.06, 0.16)	< 0.001	0.26 (0.14, 0.48)	< 0.001	7.08	-	-	
Class 2	0.24 (0.16, 0.36)	< 0.001	0.43 (0.26, 0.69)	< 0.001	4.13	-	-	
Class 3	0.26 (0.17, 0.39)	< 0.001	0.45 (0.28, 0.73)	<0.001	3.84	-	-	
Class 1+2+3	0.19 (0.14, 0.26)	< 0.001	-	-		0.39 (0.27, 0.58)	< 0.001	4.53
cerebrovascular disease	2.48 (1.73, 3.55)	< 0.001	1.79 (1.18, 2.73)	0.006	2.99	1.80 (1.18, 2.74)	0.006	3.00
CPB time (min)	1.01 (1.01, 1.01)	< 0.001	1.01 (1.00, 1.01)	< 0.001	1.09	1.01 (1.00, 1.01)	< 0.001	1.09
transfusion*	1.69 (1.54, 1.87)	< 0.001	1.28 (1.12, 1.45)	< 0.001	1.87	1.28 (1.13, 1.46)	<0.001	1.89
eGFR (ml/min/1.73m^2)	0.96 (0.95, 0.97)	< 0.001	0.98 (0.97, 0.99)	< 0.001	1.16	0.98 (0.97, 0.99)	< 0.001	1.17
age (years)	1.05 (1.03, 1.06)	<0.001	1.01 (0.99, 1.03)	0.353	1.10	1.00 (0.99, 1.03)	0.276	1.11
female, n (%)	0.83 (0.61, 1.14)	0.254	0.72 (0.49, 1.05)	0.090	2.13	0.71 (0.49, 1.05)	0.084	2.15
diabetes mellitus, n (%)	2.51 (1.80, 3.49)	<0.001	1.58 (1.07, 2.34)	0.021	2.54	1.56 (1.05, 2.30)	0.026	2.49
CKD, n (%)	8.87 (6.33, 12.43)	<0.001	2.85 (1.80, 4.53)	<0.001	5.15	2.89 (1.82, 4.59)	<0.001	5.23
MI wihtin 3 months, n (%)	3.01 (1.64, 5.51)	<0.001	1.38 (0.65, 2.94)	0.400	2.11	1.38 (0.65, 2.94)	0.397	2.11
CLD, n (%)	2.81 (1.60, 4.94)	<0.001	1.72 (0.90, 3.28)	0.102	2.82	1.72 (0.90, 3.28)	0.103	2.82
CHF, n (%)	2.50 (1.81, 3.46)	<0.001	1.46 (0.99, 2.17)	0.059	2.28	1.50 (1.01, 2.22)	0.045	2.36
LVEF (%)	0.98 (0.97, 1.00)	0.005	1.01 (0.99, 1.02)	0.365	1.09	1.01 (0.99, 1.02)	0.390	1.09
Emergency surgery, n (%)	7.59 (3.72, 15.47)	<0.001	4.08 (1.57, 10.58)	0.004	7.63	4.04 (1.56, 10.47)	0.004	7.55

CPB, cardiopulmonary bypass; eGFR, estimated glomerular filtration rate; CKR, chronic kidney disease; MI, myocardial infarction; CLD, chronic lung disease; CHF, congestive heart failure; LEVF, left ventricular ejection fraction; OR, odds ratio; CI, confidence interval; *, intraoperative erythrocyte transfusion unit

Supplementary Table S6. Logistic regression analysis of risk factors for severe AKI, excluding patients diagnosed within the first 12 postoperative hours

	Univariable analysis		Multivariable analysis (1)		Multivariable analysis (2)	
Variables	OR (95% CI)	*p*	OR (95% CI)	*p*	OR (95% CI)	*p*
Temperature trajectory						
Class 4	reference		reference		reference	
Class 1	0.16 (0.10-0.25)	<0.001	0.41 (0.25-0.67)	<0.001		
Class 2	0.23 (0.15-0.34)	<0.001	0.37 (0.23-0.60)	<0.001		
Class 3	0.28 (0.18-0.42)	<0.001	0.46 (0.29-0.72)	<0.001		
Class 1+2+3					0.41 (0.28-0.59)	<0.001
CVD	2.28 (1.59-3.25)	<0.001	1.68 (1.12-2.52)	0.012	1.70 (1.13-2.54)	0.011
EuroSCORE II	1.12 (1.09-1.15)	<0.001	1.02 (0.99-1.06)	0.142	1.03 (0.99-1.06)	0.124
CPB time(min)	1.01 (1.01-1.01)	<0.001	1.01 (1.00-1.01)	<0.001	1.01 (1.00-1.01)	<0.001
transfusion (unit) †	1.68 (1.52-1.85)	<0.001	1.24 (1.10-1.41)	<0.001	1.24 (1.09-1.41)	<0.001
eGFR (ml/min/1.73m^2)	0.97 (0.96-0.97)	<0.001	0.97 (0.97-0.98)	<0.001	0.97 (0.97-0.98)	<0.001

CVD, cerebrovascular disease; EuroSCORE, European System for Cardiac Operative Risk Evaluation; CPB, cardiopulmonary bypass; eGFR, estimated glomerular filtration rate; OR, odds ratio; CI, confidence interval; †, intraoperative erythrocyte transfusion

Supplementary Table S7. Logistic regression analysis of risk factors for severe AKI in patients with preexisting chronic kidney disease (eGFR<60 mL/min/1.73m²)

	Univariable analysis		Multivariable analysis (1)		Multivariable analysis (2)	
Variables	OR (95% CI)	*p*	OR (95% CI)	*p*	OR (95% CI)	*p*
Temperature trajectory						
Class 4	reference		reference		reference	
Class 1	0.20 (0.09-0.43)	< 0.001	0.29 (0.11-0.76)	0.012	-	-
Class 2	0.23 (0.12-0.45)	< 0.001	0.24 (0.10-0.56)	< 0.001	-	-
Class 3	0.29 (0.16-0.52)	< 0.001	0.38 (0.19-0.74)	0.004	-	-
Class 1+2+3	0.24 (0.15-0.39)	< 0.001	-	-	0.31 (0.18-0.55)	< 0.001
CVD	1.37 (0.79-2.38)	0.257	1.39 (1.71-2.72)	0.337	1.42 (0.73-2.76)	0.309
EuroSCORE II	1.04 (1.01-1.08)	0.019	0.98 (0.93–1.02)	0.287	0.98 (0.94-1.02)	0.345
CPB time (min)	1.01 (1.00-1.01)	< 0.001	1.01 (1.00–1.02)	0.004	1.01 (1.00-1.02)	0.005
transfusion†	1.45 (1.25-1.67)	< 0.001	1.24 (1.02–1.51)	0.030	1.24 (1.02-1.50)	0.033
eGFR (ml/min/1.73m²)	0.94 (0.92-0.95)	< 0.001	0.94 (0.92–0.96)	< 0.001	0.94 (0.92-0.96)	< 0.001

CVD, cerebrovascular disease; EuroSCORE, European System for Cardiac Operative Risk Evaluation; CPB, cardiopulmonary bypass; eGFR, estimated glomerular filtration rate; OR, odds ratio; CI, confidence interval; †, intraoperative erythrocyte transfusion unit

Supplementary Table S8. Logistic regression analysis of risk factors for Class 4 trajectory

	Univariable analysis		Multivariable analysis	
Variables	OR (95% CI)	*p* value	OR (95% CI)	*p* value
CVD	1.35 (1.04–1.75)	0.024	1.04 (0.79–1.38)	0.782
EuroSCORE II	1.09 (1.07–1.12)	< 0.001	1.04 (1.01–1.06)	0.003
CPB time (min)	1.002 (1.00–1.003)	0.054	0.998 (0.996–1.001)	0.199
transfusion†	1.43 (1.33–1.54)	< 0.001	1.28 (1.17–1.40)	< 0.001
eGFR (ml/min/1.73m^2)	0.98 (0.97–0.98)	< 0.001	0.980 (0.976–0.984)	< 0.001

CVD, Cerebrovascular disease; EuroSCORE, European System for Cardiac Operative Risk Evaluation; CPB, cardiopulmonary bypass; eGFR, estimated glomerular filtration rate; OR, odds ratio; CI, confidence interval; †, intraoperative erythrocyte transfusion unit

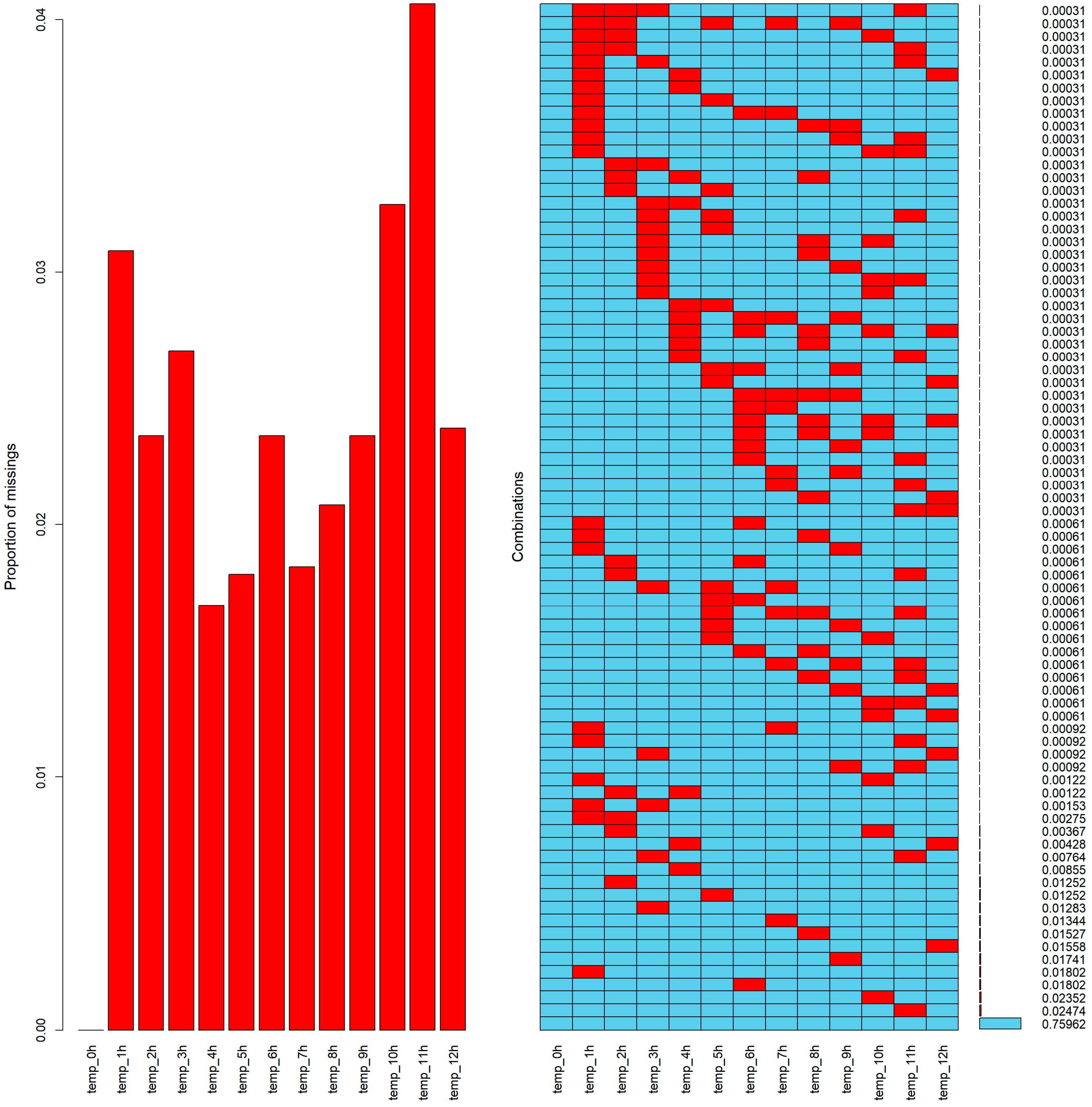

Supplementary Figure S1. Visulaization of missing data patterns

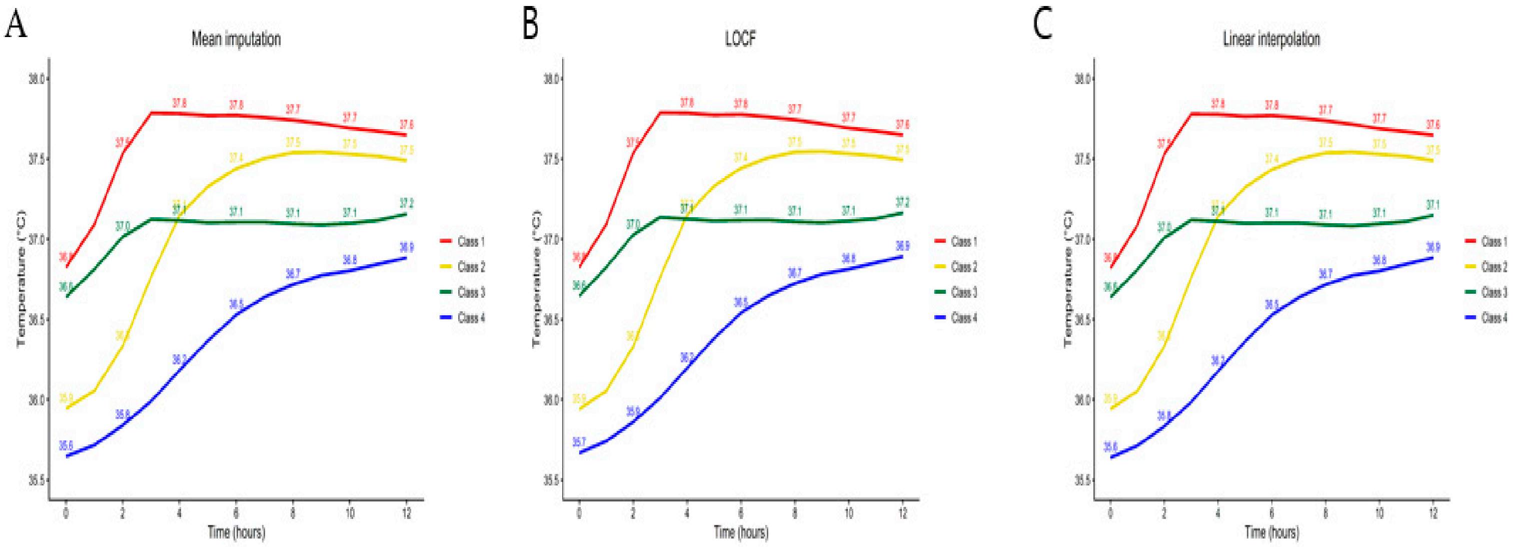

Supplementary Figure S2. Comparison of three different imputation methods for temperature data over time. (A) Mean imputation, (B) Last Observation Carried Forward (LOCF), and (C) Linear interpolation. Class 1 (red), Class 2 (yellow), Class 3 (green), and Class 4 (blue) are represented in all panels.

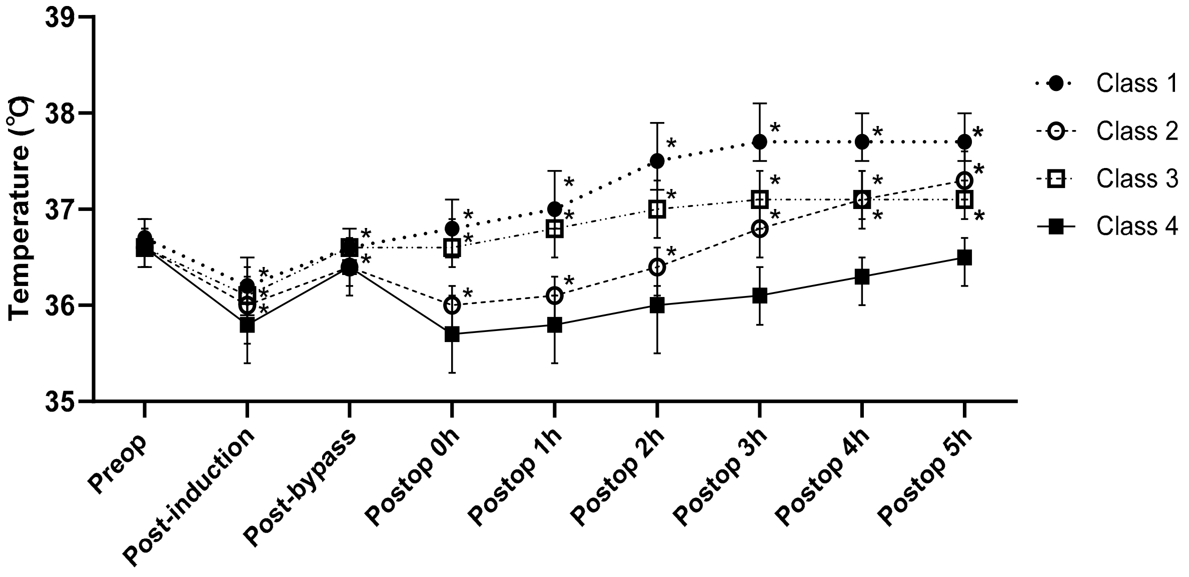

Supplementary Figure S3. Perioperative temperature changes among trajectory class

Values are median (IQR). preop, preoperative; postop, postoperative; *, $p < 0.05$ compared to Class 4
